# Comparative analysis of thrombocytopenia incidence in patients treated with generic vs. brand-name linezolid: a cohort study utilizing hospital electronic medical records

**DOI:** 10.3389/fphar.2025.1528633

**Published:** 2025-06-18

**Authors:** Zhizhou Wang, Ke Wang, Yiming Hua, Xin Hu, Xiaotong Zhang, Xiaoxi Li, Xiaoxuan Xing, Yingnan Feng, Chao Wu, Zhichao Zhang, Xianzhe Dong, Lan Zhang

**Affiliations:** ^1^ Department of Pharmacy, Xuanwu Hospital of Capital Medical University, Beijing, China; ^2^ College of Pharmacy, Zunyi Medical University, Zunyi, China

**Keywords:** Antibacterial agents, linezolid, generic drug, brand-name drug, safety, thrombocytopenia, anemia

## Abstract

**Background:**

This study aimed to evaluate the hematological safety of generic linezolid, providing data to support its rational and safe use in clinical practice.

**Methods:**

Data were collected from electronic medical records at a tertiary hospital in China between January 2019 and June 2023. We conducted a real-world, retrospective matched cohort study involving hospitalized patients treated with either generic or brand-name linezolid for bacterial infections. Propensity score matching was employed to control for potential risk factors associated with thrombocytopenia. The primary outcome was the incidence of thrombocytopenia adverse events. Secondary outcomes included rates of severe thrombocytopenia, the incidence of anemia meeting transfusion thresholds, and changes in platelet counts (PLTs) and hemoglobin (Hb) levels during follow-up.

**Results:**

A total of 218 patients received generic linezolid, while 222 patients received the brand-name version. After adjustment, each group had 137 patients. There were no significant differences in thrombocytopenia (28.44% vs. 21.17%), severe thrombocytopenia (6.42% vs. 4.95%), or anemia rates (2.75% vs. 3.15%) (P > 0.05). Similarly, reductions in PLT and HB levels during follow-up did not differ significantly (P > 0.05).

**Conclusion:**

Our results indicate no significant differences in the incidence of thrombocytopenia and severe anemia between generic and brand-name linezolid, highlighting the need for further validation in other generic formulations and diverse patient populations.

## 1 Introduction

Linezolid, an oxazolidinone antibiotic, has shown effective clinical activity against vancomycin-resistant enterococci and methicillin-resistant *Staphylococcus aureus* (Leach et al., 2011). It is widely used in hospital settings for the empiric treatment of skin and soft tissue infections, as well as hospital-acquired pneumonia and ventilator-associated pneumonia (Perry and Jarvis, 2001). However, the use of linezolid is limited by safety and tolerability concerns (Vinh and Rubinstein, 2009). Among its most significant adverse effects is myelosuppression, particularly thrombocytopenia, which has emerged as a critical safety issue that can necessitate discontinuation of therapy (Chen et al., 2012; Dong et al., 2014; González-Del Castillo et al., 2017).

Cost-effective generic drugs play a crucial role in reducing healthcare costs, and the World Health Organization (WHO) advocates for the promotion and use of high-quality generics, especially in developing countries (Kesselheim et al., 2016; Vincent, 2020). With the implementation of China’s National Centralized Drug Procurement (NCDP) policy, generic linezolid injections have increasingly replaced brand-name formulations in clinical practice. Although generic linezolid has met bioequivalence and quality standards (Bergmann et al., 2022), comprehensive studies specifically evaluating the safety and clinical equivalence of generic linezolid are lacking.

Therefore, we conducted a retrospective, matched-cohort study to evaluate the hematological safety profile of hospitalized patients treated with either generic or brand-name linezolid injections at a tertiary hospital in China. The aim of this study was to provide insights into the safety of generic linezolid, offering data that can inform its rational and safe use in clinical practice.

## 2 Materials and methods

### 2.1 Study design and population

This retrospective, single-center, observational cohort study reviewed electronic medical records (EMRs) to extract clinical information. The study included all hospitalized patients who received intravenous generic or brand-name linezolid treatment from January 2019 to June 2023, at Xuanwu Hospital, Capital Medical University. Linezolid was identified using the Anatomical Therapeutic Chemical system code J01XX08. Each patient contributed only one treatment episode to the analyses. If a patient had received linezolid during another period, only data from the first period of administration were collected.

Patients were excluded based on the following criteria: (a) younger than 18 years old, pregnant, breastfeeding, or having any of the following conditions: autoimmune diseases (e.g., systemic lupus erythematosus), solid tumors and hematologic malignancies, hypersplenism, liver cirrhosis, acute liver failure, or post-transplantation status; (b) Course of intravenous linezolid <72 h; (c) no platelet counts (PLTs) test within 72 h before linezolid treatment (baseline) or during the treatment period until 72 h after discontinuation (follow-up period), or PLTs baseline < (50 × 10^9^/L).

### 2.2 Ethics

This study was conducted in compliance with the Declaration of Helsinki and approved by the Ethics Committee of Xuanwu Hospital, Capital Medical University (2023 [156]). The requirement for informed consent was waived due to the retrospective nature of the study.

### 2.3 Exposures and follow-up

Patients dispensed generic linezolid were considered exposed; patients dispensed brand-name linezolid comprised the referent group. The first day of linezolid treatment was designated as Day 1 (D1) of the follow-up period, and the follow-up ended 72 h after discontinuation of linezolid.

### 2.4 Data collection

Patient and clinical factors were queried from EMRs. These parameters were partly chosen based on previous publications on linezolid-induced thrombocytopenia (Cattaneo et al., 2023; Zhang et al., 2023; Wang et al., 2024). The following baseline characteristics were recorded: demographics including sex, age, body height and weight; severity of illness at therapy initiation assessed using the Charlson Comorbidity Index (CCI) (Charlson et al., 1987); location of therapy initiation [intensive care unit (ICU) or non-ICU] and whether surgery was performed prior to linezolid treatment during hospitalization were also recorded. Baseline laboratory data were extracted from the most recent results within 72 h before the initiation of linezolid treatment and included neutrophil count, PLTs, Hb, serum creatinine, alanine aminotransferase, and alkaline phosphatase. Creatinine clearance was calculated using the Cockcroft–Gault equation (Cockcroft and Gault, 1976). Since our institution does not conduct therapeutic drug monitoring (TDM) for linezolid, we were unable to collect data related to linezolid blood concentrations.

During the follow-up period, the use of concomitant antibiotics, non-steroidal anti-inflammatory drugs (NSAIDs), and low molecular weight heparin (LMWH) or heparin administered for more than 72 h from the start of linezolid was recorded. Additionally, the use of thrombopoietic growth factors, erythroid growth factors, and various transfusions was documented and considered concomitant if they were administered at least once during linezolid therapy, as these interventions primarily affect PLTs and Hb. Linezolid was administered at a dosage of 0.6 g every 12 h, and the duration of linezolid therapy was recorded. Recording of complete blood count data ended 72 h after the discontinuation of linezolid therapy.

### 2.5 Outcomes

The primary outcome was thrombocytopenia, defined as any instance during the follow-up period where PLTs dropped below 150 (×10^9^/L) and there was a ≥30% decrease from PLTs baseline while on therapy (Thirot et al., 2021). The percentage change in PLTs was calculated as: (PLTs _baseline_ - lowest PLTs during follow-up)/PLTs baseline (Patel et al., 2012). For patients who developed thrombocytopenia, we assessed the time from the initiation of linezolid treatment to the onset of thrombocytopenia and evaluated its cumulative incidence over the treatment period.

Secondary outcome included: (1) severe thrombocytopenia, defined as any instance where PLTs during the follow-up period dropped below 50 (×10^9^/L) (Anthon et al., 2023); (2) distribution of baseline and lowest PLTs during follow-up. A subgroup analysis was conducted based on baseline PLTs <150 (×10^9^/L) to compare distribution across different subgroups and assess changes in PLTs. This was alculated as: (lowest PLTs during follow-up - PLTs _baseline_)/PLTs _baseline_; (3) meeting the transfusion threshold, defined as any instance where Hb during the follow-up period dropped below 70 (g/dL) and showed a ≥20% decrease from baselin Hb while on therapy (Carson et al., 2021). The percentage change in Hb was calculated as: (Hb _baseline_ - lowest Hb during follow-up)/Hb _baseline_; (4) distribution of baseline and lowest Hb during follow-up. A subgroup analysis was conducted for baseline Hb < 110 (g/dL) to compare distribution across different subgroups and assess Hb changes. The calculated for Hb change was: (lowest Hb during follow-up - Hb _baseline_)/Hb _baseline_.

### 2.6 Statistical analysis

All statistical analyses were performed using R version 4.3.2. To balance baseline differences, propensity scores for the likelihood of receiving linezolid were estimated using logistic regression, accounting for all prespecified parameters. Propensity score matching (PSM) was performed using a 1:1 nearest-neighbor approach with a maximum caliper of 0.2. Treatment effects were evaluated without further adjustment, as all covariates were balanced in the matched cohort.

Continuous variables were tested for normality using the Shapiro-Wilk test. Non-normally distributed variables were presented as medians and interquartile range (IQR) and compared between groups using the Wilcoxon Rank-Sum Test. Categorical variables were expressed as frequencies and percentages and compared using the Chi-Square Test. To assess the risk of hematological adverse events, binary logistic regression analysis was employed. The odds ratio (OR) and 95% confidence interval (CI) were calculated to quantify the association between the use of generic versus brand-name linezolid.

The Kaplan-Meier method was used to estimate the time to thrombocytopenia onset for generic and brand-name linezolid, and the log-rank test was used to compare the cumulative incidence of thrombocytopenia. Univariate Cox proportional hazards regression was performed to compare the time to thrombocytopenia onset between generic and brand-name linezolid before and after PSM. A p-value of less than 0.05 was considered statistically significant.

## 3 Results

### 3.1 Patient demographic and clinical characteristics

**FIGURE 1:**
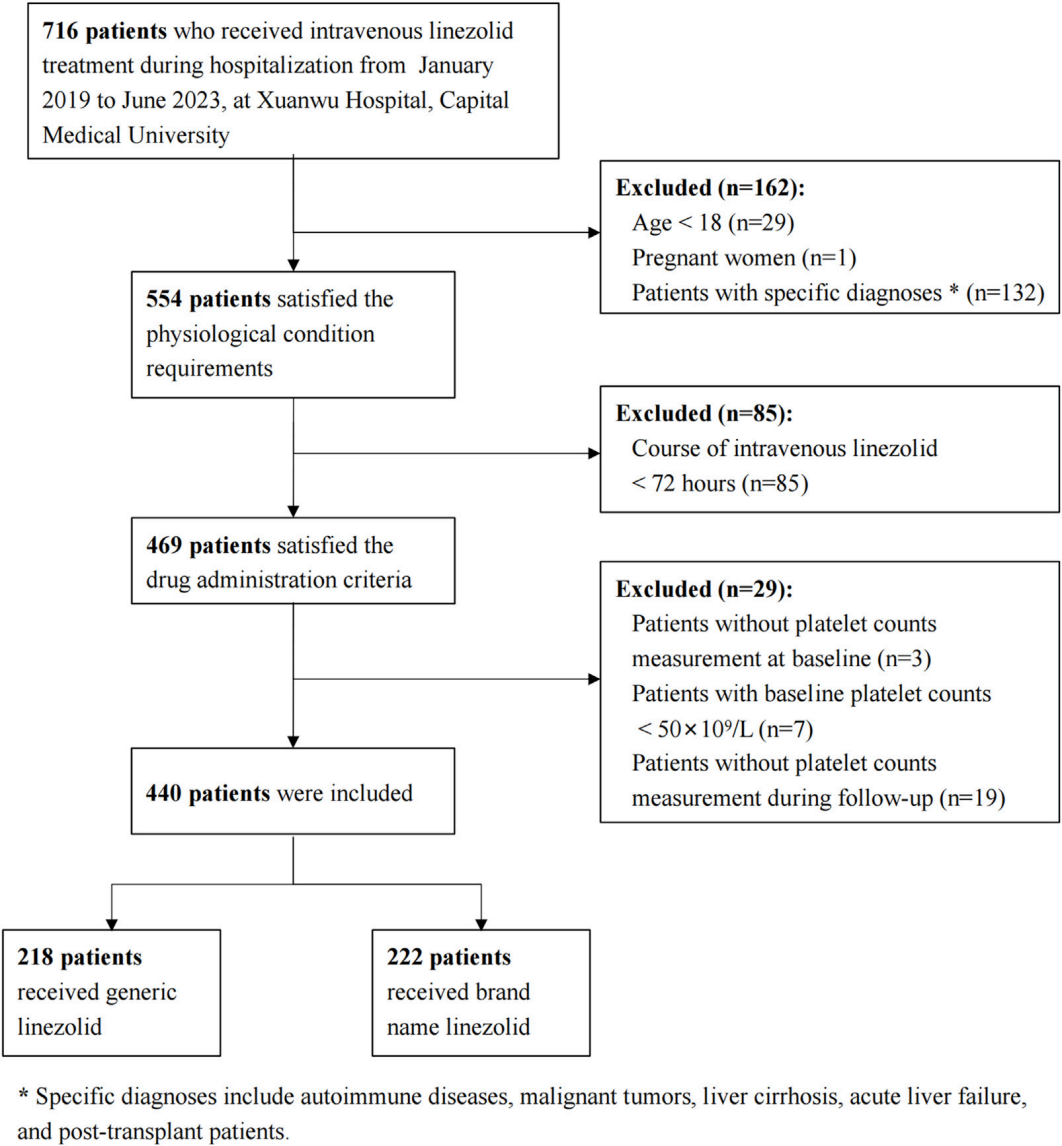
Flowchart of the study inclusion and exclusion process.

A total of 716 patients who received linezolid treatment during the study period were initially identified. A total of 276 patients were excluded due to physiological and pathological conditions, treatment duration less than 72 h, or issues with PLTs measurement. This resulted in a final cohort of 440 patients, with 218 receiving generic linezolid and 222 receiving brand-name linezolid (Figure 1).

In the original cohort, several covariates showed significant differences between the two groups. The median creatinine clearance was 71.20 mL/min (IQR: 33.72–139.92) in the generic linezolid group compared to 99.33 mL/min (IQR: 53.21–138.42) in the brand-name linezolid group (p = 0.025). The median PLTs was lower in the generic linezolid group than in the brand-name linezolid group (197.50 vs. 234.50 (×10^9^/L), p = 0.028). Similarly, Hb levels were significantly lower in the generic linezolid group (100.00 vs. 108.00 (g/dL), p < 0.001). A higher percentage of patients in the brand-name linezolid group were using antibiotics (79.3% vs. 51.4%, p < 0.001) and erythroid growth factors (7.2% vs. 1.8%, p = 0.013). Conversely, a higher percentage of patients in the generic linezolid group were using LMWH or heparin (30.3% vs. 19.8%, p = 0.015). After PSM, 137 pairs were used for the final analysis. All the demographic and clinical characteristics were balanced between the two groups (Table 1).

**TABLE 1:** Baseline demographic and clinical characteristics of the Original cohort and the PSM cohort.

Variables	Original cohort			PSM cohort		
	Generic linezolid (n = 218)	Brand-name linezolid (n = 222)	P-value	Generic linezolid (n = 137)	Brand-name linezolid (n = 137)	P-value
Male	143 (65.6)	146 (65.8)	1.000	90 (65.7)	97 (70.8)	0.436
Age (years)			0.089			0.841
40–64	97 (44.5)	85 (38.3)		57 (41.6)	58 (42.3)	
18–39	35 (16.1)	24 (10.8)		21 (15.3)	16 (11.7)	
65–79	50 (22.9)	64 (28.8)		32 (23.4)	35 (25.5)	
≥80	36 (16.5)	49 (22.1)		27 (19.7)	28 (20.4)	
Body mass index (kg/m^2^)			0.186			0.994
18.5–24.9	98 (45.0)	118 (53.2)		63 (46.0)	64 (46.7)	
<18.5	18 (8.3)	18 (8.1)		13 (9.5)	14 (10.2)	
25–29.9	71 (32.6)	67 (30.2)		43 (31.4)	42 (30.7)	
≥30	31 (14.2)	19 (8.6)		18 (13.1)	17 (12.4)	
CCI score	1.00 (0.00–3.00)	1.00 (0.00–3.00)	0.156	1.00 (0.00–3.00)	1.00 (0.00–3.00)	0.652
ICU	153 (70.2)	139 (62.6)	0.114	91 (66.4)	89 (65.0)	0.899
Surgery	107 (49.1)	117 (52.7)	0.507	71 (51.8)	72 (52.6)	1.000
Duration of linezolid treatment (days)			0.581			0.991
7–13	99 (45.4)	95 (42.8)		62 (45.3)	63 (46.0)	
3–6	87 (39.9)	99 (44.6)		53 (38.7)	52 (38.0)	
≥14	32 (14.7)	28 (12.6)		22 (16.1)	22 (16.1)	
Baseline biological parameters						
Neutrophil counts (×10^9^/L)	8.70 (5.44–11.96)	7.83 (5.24–12.19)	0.575	8.80 (5.76–12.48)	8.28 (5.82–12.83)	0.788
PLTs (×10^9^/L)	197.50 (147.25–287.00)	234.50 (165.00–299.50)	0.028	216.00 (157.00–309.00)	220.00 (154.00–298.00)	0.991
Hb (g/dL)	100.00 (87.00–114.00)	108.00 (93.00–123.00)	<0.001	105.00 (91.00–118.00)	103.00 (91.00–120.00)	0.990
Alanine aminotransferase (U/L)	24.00 (13.00–49.00)	22.50 (14.00–46.75)	0.856	25.00 (14.00–57.00)	28.00 (17.00–53.00)	0.760
Alkaline phosphatase (U/L)	73.00 (58.00–112.00)	75.00 (60.00–101.00)	0.892	73.00 (58.00–106.00)	80.00 (62.00–111.00)	0.190
Creatinine clearance (mL/min)	71.20 (33.72–139.92)	99.33 (53.21–138.42)	0.025	82.33 (40.75–140.73)	101.71 (52.02–139.24)	0.124
Combined treatment						
NSAIDs	32 (14.7)	43 (19.4)	0.237	24 (17.5)	19 (13.9)	0.506
Antibiotics	112 (51.4)	176 (79.3)	<0.001	90 (65.7)	93 (67.9)	0.798
LMWH or heparin	66 (30.3)	44 (19.8)	0.015	34 (24.8)	35 (25.5)	1.000
Thrombopoietic growth factors	8 (3.7)	6 (2.7)	0.759	5 (3.6)	3 (2.2)	0.720
Erythroid growth factors	4 (1.8)	16 (7.2)	0.013	4 (2.9)	6 (4.4)	0.747
Platelet transfusion	23 (10.6)	14 (6.3)	0.152	11 (8.0)	9 (6.6)	0.816
Red blood cells transfusion	90 (41.3)	79 (35.6)	0.258	51 (37.2)	54 (39.4)	0.804
Fresh frozen plasma transfusion	70 (32.1)	63 (28.4)	0.454	42 (30.7)	50 (36.5)	0.371

Values are presented as number (percent of within group) or median (interquartile range).

P-value obtained from chi-square analysis for categorical variables and the Wilcoxon rank-sum test for continuous variables. P < 0.05 considered statistically significant.

PSM, propensity score matching; CCI, charlson comorbidity index; ICU, intensive care unit; PLTs, platelet counts; Hb, hemoglobin; NSAIDs, non-steroidal anti-inflammatory drugs; LMWH, low molecular weight heparin.

### 3.2 Risk of thrombocytopenia adverse events

#### 3.2.1 Thrombocytopenia incidence

During the follow-up period, 62 patients (28.44%) in the generic linezolid group experienced thrombocytopenia, compared to 47 patients (21.17%) in the brand-name linezolid group. The unadjusted OR was 1.48 (95% CI: 0.96–2.30, P = 0.078). After adjusting for potential confounders using PSM, the adjusted OR was 1.32 (95% CI: 0.59–3.03, P = 0.507).

The incidence of severe thrombocytopenia was 6.42% (14 patients) in the generic linezolid group and 4.95% (11 patients) in the brand-name linezolid group. The unadjusted OR for severe thrombocytopenia was 1.12 (95% CI: 0.66–1.90, P = 0.684), suggesting no significant difference between the groups. After adjusting for potential confounders, the adjusted OR was 0.87 (95% CI: 0.30–2.49, P = 0.791), which also indicated no significant difference between the groups (Table 2).

**TABLE 2:** Risk of hematological adverse events.

Hematological adverse event	Generic linezolid	Brand-name linezolid	Unadjusted		PSM adjusted	
	(n = 218)	(n = 222)	OR (95% CI)	P-value	OR (95% CI)	P-value
Thrombocytopenia	62 (28.44)	47 (21.17)	1.48 (0.96–2.30)	0.078	1.32 (0.59–3.03)	0.507
Severe thrombocytopenia	14 (6.42)	11 (4.95)	1.12 (0.66–1.90)	0.684	0.87 (0.30–2.49)	0.791
Transfusion threshold	6 (2.75)	7 (3.15)	0.87 (0.28–2.66)	0.804	0.83 (0.23–2.81)	0.759

Binary logistic regression analysis was performed.

CI, confidence interval; OR, odds ratio.

#### 3.2.2 Median time to onset and cumulative incidence

**FIGURE 2:**
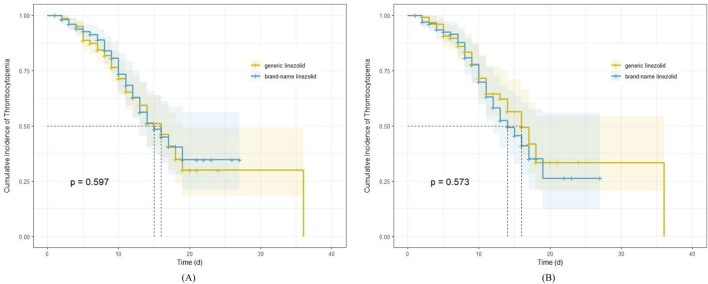
Kaplan-Meier survival estimates for the impact of generic and brand-name linezolid on cumulative incidence of thrombocytopenia; **(A)** before propensity score analyses; **(B)** after propensity score analyses.

The median time to onset of thrombocytopenia was 16 days in the generic linezolid group, both before and after adjustment. In the brand-name linezolid group, the median time to onset was 15 days before adjustment and 14 days after adjustment. Kaplan-Meier survival estimates indicated no significant difference in the incidence of thrombocytopenia between the two groups (Figure 2), both before and after adjustment (log-rank test, P = 0.597 and P = 0.573, respectively).

#### 3.2.3 PLTs following linezolid treatment

In the PSM cohort, the median lowest PLTs during follow-up were 163.00 (×10^9^/L) in the generic linezolid group and 172.00 (×10^9^/L) in the brand-name linezolid group (P = 0.413). No statistically significant differences were observed in the distribution of PLTs between the two groups, both at baseline and during follow-up. When stratified by baseline PLTs with a cutoff of 150 (×10^9^/L), subgroup analyses revealed no significant differences in the distribution of baseline and follow-up PLTs between the generic and brand-name linezolid groups, regardless of baseline PLTs levels (all P-values >0.05) (Table 3).

**TABLE 3:** Characteristics of PLTs changes in patients treated with generic and brand-name linezolid.

PLTs (×10^9^/L)		Generic linezolid	Brand-name linezolid	P-value
Overall (n = 137)	Baseline	216.00 (157.00–309.00)	220.00 (154.00–298.00)	0.992
	Lowest during follow-up	163.00 (98.50–246.50)	172.00 (107.00–262.00)	0.413
Baseline PLTs <150 (n = 31)	Baseline	114.00 (93.00–137.00)	113.00 (78.00–132.00)	0.346
	Lowest during follow-up	93.00 (62.00–116.00)	100.00 (50.00–131.00)	0.980
Baseline PLTs ≥150 (n = 106)	Baseline	246.50 (195.25–348.50)	253.50 (200.00–335.50)	0.893
	Lowest during follow-up	189.50 (126.25–267.25)	196.00 (142.75–290.75)	0.327

Values are presented as median (interquartile range). P-value obtained from the Wilcoxon rank-sum test.

PLTs, platelet counts.

#### 3.2.4 Subgroup analysis of PLTs changes following linezolid treatment

**FIGURE 3:**
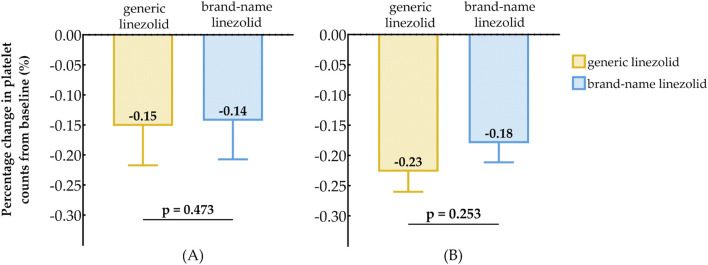
Comparison of Baseline Reduction in Platelet Counts After Treatment Between Generic Linezolid Group and Brand-Name Linezolid Group; **(A)** Baseline PLT < 150×10^9^/L; **(B)** Baseline PLT ≥ 150×10^9^/L.

For patients with baseline PLTs <150 (×10^9^/L), the median percentage change in PLTs from baseline was −15% in the generic linezolid group and −14% in the brand-name linezolid group (Figure 3A), with no statistically significant difference (P = 0.473). Similarly, for patients with baseline PLTs ≥150 (×10^9^/L), the median percentage change was −23% in the generic linezolid group and −18% in the brand-name linezolid group (Figure 3B), also showing no significant difference (P = 0.253).

### 3.3 Risk of anemia adverse events

#### 3.3.1 Meeting the transfusion threshold incidence

Meeting the transfusion threshold occurred in 2.75% (6 patients) of the generic linezolid group and 3.15% (7 patients) of the brand-name linezolid group. The unadjusted OR was 0.87 (95% CI: 0.28–2.66, P = 0.804), and the adjusted OR was 0.83 (95% CI: 0.23–2.81, P = 0.759), showing no significant difference (Table 2).

#### 3.3.2 Hb following linezolid treatment

In the PSM cohort, the median lowest Hb during follow-up were 91.00 [79.50, 101.50] (g/dL) in the generic linezolid group and 88.00 [77.00, 110.00] (g/dL) in the brand-name linezolid group (P = 0.838). Hb levels during follow-up did not differ significantly between the two groups.

Further analysis, stratified by baseline Hb with a cutoff of 110 g/dL, showed no significant difference in Hb distribution at both baseline and follow-up for patients with a baseline Hb < 110 g/dL (P > 0.05). For patients with a baseline Hb ≥ 110 g/dL, the median baseline Hb was similar between the two groups. However, during follow-up, a significant difference emerged: the median lowest Hb for the generic group was 106.00 [93.00, 117.00] (g/dL), which was lower than the 116.50 [96.25, 123.75] (g/dL) seen in the brand-name group, with this difference reaching statistical significance (P = 0.043) (Table 4).

**TABLE 4:** Characteristics of Hb changes in patients treated with generic and brand-name linezolid.

Hb (g/dL)		Generic linezolid	Brand-name linezolid	P-value
Overall (n = 137)	Baseline	105.00 (91.00–118.00)	103.00 (91.00–120.00)	0.990
	Lowest during follow-up	91.00 (79.50–101.50)	88.00 (77.00–110.00)	0.838
Baseline Hb < 110 (n = 82:81)	Baseline	94.00 (81.75–102.25)	93.00 (86.00–99.50)	0.647
	Lowest during follow-up	83.00 (73.75–93.00)	81.00 (73.00–89.00)	0.424
Baseline Hb ≥ 110 (n = 55:56)	Baseline	121.00 (115.00–137.00)	123.00 (116.00–134.75)	0.719
	Lowest during follow-up	106.00 (93.00–117.00)	116.50 (96.25–123.75)	0.043

Values are presented as median (interquartile range). P-value obtained from the Wilcoxon rank-sum test.

Hb, hemoglobin.

#### 3.3.3 Subgroup analysis of Hb changes following linezolid treatment

**FIGURE 4:**
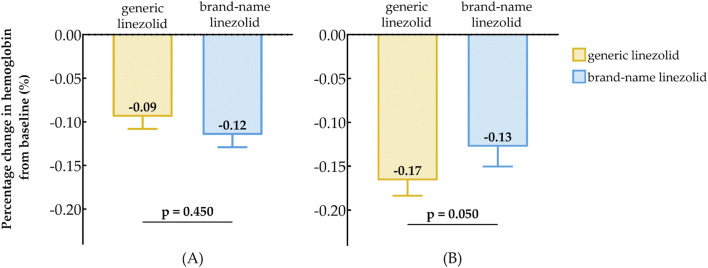
Comparison of Baseline Reduction in hemoglobin After Treatment Between Generic Linezolid Group and Brand-Name Linezolid Group; **(A)** Baseline Hb < 110 g/L; **(B)** Baseline Hb ≥ 110 g/L.

For patients with baseline HB < 110 (g/dL), the median percentage change in Hb from baseline was −9% in the generic linezolid group and −12% in the brand-name linezolid group (Figure 4A), with no statistically significant difference between the two groups (P = 0.450). For patients with baseline HB ≥ 110 (g/dL), the median percentage change in Hb was −17% in the generic linezolid group, which was greater than the −13% change observed in the brand-name linezolid group (Figure 4B). The difference between the two groups approached statistical significance (P = 0.050).

## 4 Discussion

Linezolid is generally well-tolerated, but hematological toxicity—primarily thrombocytopenia and anemia—remains a significant adverse drug reaction that can severely affect clinical outcomes when severe (Han et al., 2022; Zou et al., 2024). Numerous studies have reported thrombocytopenia following linezolid treatment (Zhang et al., 2023; Inoue et al., 2023), comparing its safety profile with vancomycin (Al-Harbi et al., 2022) and other antibiotics used for multi-drug-resistant Gram-positive infections (Ju et al., 2024; Yu et al., 2024). Despite this, little attention has been given to the safety of generic linezolid, and no studies have specifically addressed the risk of hematological adverse events associated with generic formulations. To our knowledge, this study is the first to use EMRs to compare the incidence of hematological toxicity, particularly thrombocytopenia, between generic and brand-name linezolid.

We reviewed EMRs of all patients treated with linezolid injection at a tertiary comprehensive medical institution in China over a 5-year period. To minimize the impact of secondary thrombocytopenia, we excluded patients (Takahashi et al., 2018; Scharf, 2021; Holden et al., 2023; Kashiwagi, 2023; Bussel and Knightly, 2024) who were pregnant, had immunosuppressive conditions (such as systemic lupus erythematosus, multiple sclerosis, or post-transplant status), hematologic malignancies, malignant solid tumors, cirrhosis, or acute and chronic liver failure. Baseline PLTs has been identified as a risk factor for thrombocytopenia (Cazavet et al., 2020). Therefore, patients with a baseline PLTs <50 (×10^9^/L) before linezolid treatment were also excluded, as the risk of bleeding significantly increases when PLTs <50 (×10^9^/L) (Napolitano et al., 2019). This threshold is commonly used for platelet transfusion prior to surgery or invasive procedures (Kumar et al., 2015). Given this, subsequent PLTs changes may be less influenced by linezolid. Ultimately, we observed hematological changes in 440 patients treated with either generic or brand-name linezolid.

In this study, the incidence of thrombocytopenia was 28.44% in patients receiving generic linezolid and 21.17% in those receiving brand-name linezolid, with no statistically significant differences between the groups before and after PSM adjustment. Early clinical trials reported a low incidence of thrombocytopenia (<3% (Gerson et al., 2002)) following linezolid treatment. However, post-marketing retrospective or prospective studies have reported a much higher and widely variable incidence, ranging from 7.4% to 64.7% (Takahashi et al., 2021). This discrepancy may be attributed to the varying definitions of thrombocytopenia across studies. Previous studies often defined thrombocytopenia solely based on a fixed post-treatment PLTs threshold, commonly <100 (×10^9^/L) (U.S. Department of Health and Human Services NIoH, 2017), without adequately considering the impact of baseline PLTs on outcomes. In contrast, our study assessed thrombocytopenia using both a following treatment PLTs <150 (×10^9^/L) and a ≥30% decrease from baseline, providing a more comprehensive and objective evaluation.

Severe thrombocytopenia, defined as a PLTs <50 (×10^9^/L) during follow-up, was observed in 2.75% of patients in the generic linezolid group and 3.15% in the brand-name group, with no significant differences before or after PSM adjustment. These findings are consistent with those reported by Nimish Patel in the Upstate New York VA Healthcare Network study (Patel et al., 2012), which found a 3.6% incidence of severe thrombocytopenia among patients treated with linezolid compared to vancomycin for ≥48 h, with matching for factors such as age, ICU status, and baseline platelet levels.

Recent research indicated that baseline PLT were associated with an increased risk of thrombocytopenia (Inoue et al., 2024). Our subgroup analysis, stratified by baseline PLTs with a cutoff of 150 (×10^9^/L), found no significant differences in nadir PLTs or the extent of PLTs changes during follow-up between the generic and brand-name linezolid groups. The reduction in PLTs ranged from 14% to 23%. These findings suggest that the type of linezolid, whether generic or brand-name, does not significantly affect the extent of PLTs reduction, regardless of baseline PLTs.

In our study, the incidence of grade 4 potentially life-threatening anemia (U.S. Department of Health and Human Services NIoH, 2017), defined as meeting the transfusion threshold, was 2.75% in the generic linezolid group and 3.15% in the brand-name group, with no significant difference observed between the groups before and after adjustment. A prospective observational study of 151 patients with tuberculosis treated with linezolid reported a similar incidence of grade 4 anemia at 3.97% (6/151) during the observation period (Pratama et al., 2021), which is consistent with our findings.

In our PSM cohort, no significant differences in the lowest Hb were observed during follow-up. Previous studies have produced inconsistent findings regarding the relationship between baseline Hb and the development of anemia in patients receiving linezolid treatment (Senneville et al., 2004; Qin et al., 2021). In our study, among patients with baseline Hb ≥ 110 (g/dL), the decrease in Hb during follow-up was significantly greater in the generic linezolid group, with a median difference of 10 (g/dL) compared to the brand-name group. This warrants further investigation into the association between baseline Hb and anemia (Ma et al., 2024). It is important to note that Hb are influenced by multiple factors, including the control of systemic infections, surgical interventions, liver and kidney function, and supportive measures such as transfusions or erythropoietin administration. These factors may modulate bone marrow function during linezolid treatment, potentially enhancing or diminishing its myelosuppressive effects (Veerman et al., 2023).While this study primarily focused on thrombocytopenia, other potential confounding factors influence Hb may not have been fully adjusted for. Therefore, the impact of generic linezolid on Hb should be further investigated in future studies.

The clinical efficacy and safety of generic drugs are critical factors influencing patient outcomes. In previous studies comparing generic versus brand-name antimicrobial agents, the focus has typically been on drug quality standards (Hambisa et al., 2019; Mwalwisi et al., 2024), *in vitro* drug susceptibility (i.e., microbiological activity) (Akhi et al., 2014; Avianto et al., 2020), pharmacokinetic properties (Mer et al., 2016; Amran et al., 2021; Lv et al., 2021)and clinical efficacy (Lin et al., 2017; Machado-Alba et al., 2018; Garnica-Velandia et al., 2021). However, limited attention has been given to the safety consistency between generic and brand-name antimicrobial agents (Sutton et al., 2015). Safety outcomes are often reported as secondary endpoints in clinical efficacy studies, which may lead to insufficient statistical power to adequately assess and compare potential safety differences.

The strengths of this study lie in its use of real-world EMRs, which provides objective outcome measures and ensures the inclusion of high-risk populations often excluded from clinical trials enhancing the generalizability of the results. Additionally, the use of PSM helped adjust for potential confounding biases, thoroughly accounting for relevant risk factors affecting PLTs. Furthermore, the stratified analysis based on baseline PLTs demonstrated that there was no significant difference in the effect of generic linezolid on PLTs compared to the brand-name drug.

However, this study has several limitations. First, as an observational study based on retrospective data, there may be unmeasured residual confounding factors. Second, linezolid is currently administered as a fixed dose of 600 mg every 12 h to all patients, we used the duration of linezolid treatment as a proxy for therapy exposure, but we were unable to perform TDM. Previous research suggested that prospective TDM may help prevent linezolid-induced thrombocytopenia (Komatsu et al., 2022; Lin et al., 2022). Third, we excluded patients who received treatment for less than 72 h or lacked hematological data. In clinical practice, patients on short courses of linezolid may not routinely undergo hematological monitoring. Similar exclusions have been made in other observational studies due to missing laboratory data (Bai et al., 2022). Finally, this study evaluated the efficacy and safety of only one generic linezolid formulation, which may not fully reflect the effects of other generic linezolid products.

## 5 Conclusion

To summarize, this study demonstrates that the hematological toxicity of generic linezolid is consistent with that of the brand-name formulation in routine clinical practice. There are no significant differences between generic and brand-name linezolid in terms of their effects on PLTs, both in terms of thrombocytopenia incidence and the extent of platelet reduction. This suggests that generic linezolid and brand-name linezolid can be used interchangeably with regard to their impact on PLTs, offering clinicians confidence in prescribing generic formulations without compromising patient safety.

## Data Availability

The data analyzed in this study is subject to the following licenses/restrictions: The datasets are not publicly available due to privacy or ethical restrictions but are available from the corresponding authors on reasonable request. Requests to access these datasets should be directed to Xianzhe Dong, dongxianzhe@xwhosp.org.
